# Complete Genome Sequences of Three Polynucleobacter sp. Subcluster PnecC Strains, KF022, KF023, and KF032, Isolated from a Shallow Eutrophic Lake and a River in Japan

**DOI:** 10.1128/mra.01234-22

**Published:** 2023-02-16

**Authors:** Yusuke Ogata, Keiji Watanabe, Shusuke Takemine, Chie Shindo, Rina Kurokawa, Wataru Suda

**Affiliations:** a RIKEN Center for Integrative Medical Sciences, Yokohama, Kanagawa, Japan; b Center for Environmental Science in Saitama, Kazo, Saitama, Japan; University of Southern California

## Abstract

The genus *Polynucleobacter* subcluster PnecC consists of bacteria representing the ubiquitous taxon of freshwater bacterioplankton. Here, we report the complete genome sequences of three *Polynucleobacter* sp. (PnecC) strains, namely, KF022, KF023, and KF032, which were isolated from surface water of a temperate shallow eutrophic lake and its inflow river in Japan.

## ANNOUNCEMENT

The free-living freshwater bacterioplankton of the genus *Polynucleobacter* that belong to subcluster PnecC represent one of the most well-known bacterial taxa in worldwide distribution (1). All PnecC-affiliated strains share 16S rRNA gene sequence similarities of >99%. However, phenotypic and genotypic differences within PnecC have been reported (2). Furthermore, within PnecC, phylogeographic separation related to climatic division has been observed (1). Further accumulation of genomic information might lead to elucidation of the phylogeographic fine resolution of ubiquitous PnecC taxa.

Here, we report the whole-genome sequences of three *Polynucleobacter* sp. subcluster PnecC strains, namely, KF022 (JCM 16613), KF023 (JCM 16614), and KF032 (JCM 16615), which were isolated from samples collected from the surface water (depth of 0 to 50 cm) of Lake Kasumigaura (a temperate shallow eutrophic lake), Itako, Ibaraki, Japan (35°54′58″N, 140°36′05″E), and Sakura River, Tsukuba, Ibaraki, Japan (36°06′47″N, 140°08′39″E), on 30 June 2006. The 10-mL samples were filtered through a disposable syringe equipped with a 0.7-μm particle-retention glass-fiber filter (Puradisc 25 GF/F; Whatman, Germany). After filtration, 100-μL aliquots of filtrates were spread on modified Reasoner’s 2A (MR2A) agar plates and incubated at 25°C for 2 to 7 days (3). One bacterial colony for each strain was inoculated into sterile MR2A liquid medium (pH 7.2) and incubated at 25°C for 2 days with reciprocal shaking (120 rpm), and the pure strain cell suspensions were preserved at −80°C as stocks in MR2A broth supplemented with 20% (wt/vol) glycerol. Cells from the glycerol stocks were inoculated and cultured in MR2A liquid medium, harvested by centrifugation, and used for genomic DNA extraction.

Genomic DNA was extracted from strains KF022, KF023, and KF032 using enzymatic lysis and phenol-chloroform-isoamyl alcohol extraction as described previously (4). Whole-genome sequencing was performed using a Sequel II system (Pacific Biosciences [PacBio], USA). The library was prepared with the SMRTbell Express template preparation kit v.2.0 (PacBio), with DNA shearing with g-TUBEs (Covaris, USA) and with 10- to 15-kb target lengths. The PacBio reads were converted to circular consensus sequencing (CCS) reads using CCS software v.6.2.0 (https://github.com/PacificBiosciences/ccs) and assembled using the assembler Canu v.2.1.1 with specified parameters (minReadLength=2200, minOverlapLength=2200) (5), and the generated contig was checked for circularization to remap the CCS reads by minimap2 v.2.24-r1122 (6). The genomic sequences obtained were annotated and rotated to start at *dnaA* using DFAST (https://dfast.nig.ac.jp) (7). Default parameters were used for all software analyses unless otherwise specified. The reads obtained and the genomic sequences generated are summarized in Table 1. The average nucleotide identity by orthology (OrthoANI) and the average amino acid identity (AAI) between strains KF022 and Polynucleobacter yangtzensis MWH-JaK3^T^ (PnecC) were 87.08% and 93.44%, respectively, those between strains KF023 and P. yangtzensis MWH-JaK3^T^ were 96.25% and 96.61%, respectively, and those between strains KF032 and P. yangtzensis MWH-JaK3^T^ were 96.41% and 97.01%, respectively. Thus, strains KF022, KF023, and KF032 belong to *Polynucleobacter* sp. subcluster PnecC.

**TABLE 1:** Summarized data on reads and contigs obtained for *Polynucleobacter* sp. (PnecC) strains KF022, KF023, and KF032

Parameter	Data for strain:		
	KF022	KF023	KF032
Habitat	Sakura River	Sakura River	Lake Kasumigaura
Isolation source	Surface water	Surface water	Surface water
No. of quality-passed Sequel reads	30,000	30,000	30,000
Total size of quality-passed Sequel reads (bp)	481,055,455	417,706,099	408,705,851
Quality-passed Sequel read *N*_50_ (bp)	16,082	13,784	13,509
No. of coding sequences	1,881	1,936	1,889
Total no. of contigs	1	1	1
Circular	Yes	Yes	Yes
BioProject accession no.	PRJDB14432	PRJDB14432	PRJDB14432
BioSample accession no.	SAMD00546904	SAMD00546905	SAMD00546906
SRA accession no.	DRR413267	DRR413268	DRR413269
Genome size (bp)	1,868,546	1,976,099	1,930,827
GC content (%)	45.5	45.5	45.6
GenBank/ENA/DDBJ accession no.	AP026972	AP026973	AP026974

Based on annotation results for the strain KF022 and KF023 genome sequences, the strains contained a gene predicted to encode polyphosphate kinase 2 (*ppk2*), which is associated with the intracellular accumulation of polyphosphate, whereas strain KF032 was lacking the gene. Furthermore, the three strains contained a gene encoding a putative alanine dehydrogenase-like protein. In addition, only strain KF023 possessed a gene encoding a glutamate dehydrogenase-like protein. All strains lacked a gene encoding a putative rhodopsin-like protein.

### Data availability.

The chromosome sequences and reads for strains KF022, KF023, and KF032 were deposited in the GenBank/ENA/DDBJ database, and the details are summarized in Table 1.
